# Seeing the Future: A Review of Ocular Therapy

**DOI:** 10.3390/bioengineering11020179

**Published:** 2024-02-13

**Authors:** Maiya Whalen, Monica Akula, Shannon M. McNamee, Margaret M. DeAngelis, Neena B. Haider

**Affiliations:** 1 Department of Biology, Boston College, Chestnut Hill, MA 02467, USA; 2 Shifa Precision, Boston, MA 02138, USA; 3 Department of Ophthalmology, Jacobs School of Medicine & Biomedical Sciences, University at Buffalo, Buffalo, NY 14203, USA; 4 Department of Cell Biology, Harvard Medical School, Boston, MA 02138, USA

**Keywords:** ocular therapy, gene therapy, cell therapy, retinal prosthesis, nanoparticles

## Abstract

Ocular diseases present a unique challenge and opportunity for therapeutic development. The eye has distinct advantages as a therapy target given its accessibility, compartmentalization, immune privilege, and size. Various methodologies for therapeutic delivery in ocular diseases are under investigation that impact long-term efficacy, toxicity, invasiveness, and delivery range. While gene, cell, and antibody therapy and nanoparticle delivery directly treat regions that have been damaged by disease, they can be limited in the duration of the therapeutic delivery and have a focal effect. In contrast, contact lenses and ocular implants can more effectively achieve sustained and widespread delivery of therapies; however, they can increase dilution of therapeutics, which may result in reduced effectiveness. Current therapies either offer a sustained release or a broad therapeutic effect, and future directions should aim toward achieving both. This review discusses current ocular therapy delivery systems and their applications, mechanisms for delivering therapeutic products to ocular tissues, advantages and challenges associated with each delivery system, current approved therapies, and clinical trials. Future directions for the improvement in existing ocular therapies include combination therapies, such as combined cell and gene therapies, as well as AI-driven devices, such as cortical implants that directly transmit visual information to the cortex.

## 1. Background

Vision impairment is a life-altering condition that affects approximately 2.2 billion people worldwide. Among the top five causes of vision impairment or blindness are age-related macular degeneration (AMD), glaucoma, and diabetic retinopathy, which affect almost 20 million people globally [1,2]. Additionally, rare blindness diseases such as retinitis pigmentosa (RP) affect about 1.5 million people worldwide [3]. In recent years, many ocular therapy delivery techniques and modalities have emerged to try to meet the need for effective treatments of both common and rare ocular diseases. The eye presents a unique target for therapeutic delivery and treatment given its accessibility, size, and compartmentalization [4]. It also has an immune-privileged status, and thus may be uniquely suited to accommodate the antigenicity of therapeutic delivery systems [5,6]. Both the cornea and the blood–retinal barrier are essential to the maintenance of the eye as an immune-privileged site, meaning that the introduction of foreign substances is much less likely to cause an inflammatory response. Additionally, the eye’s small and confined structure reduces the required dosage of therapeutics and reduces the risk of systemic spread of the locally administered therapy, which mitigates the risk of potential immune responses [7,8]. Furthermore, the eye is easily accessible for surgical interventions and diagnostic tests, making therapeutic administration and subsequent monitoring relatively simple.

Currently, four main types of treatment modalities are under investigation for their potential efficacy and safety in various ocular conditions such as AMD, RP, diabetic retinopathy, uveitis, glaucoma, cataracts, dry eye, and corneal opacification. Gene therapy, cell therapy, antibody therapy, and ocular prostheses show great promise as therapeutic treatments. Gene therapies target and alter disrupted gene function in conditions with identified genetic causes, often with the aid of a vector [7,9]. Cell therapies show potential as a therapeutic by utilizing stem cells to restore or replace dysfunctional cells in the retina; however, none have gone beyond clinical trials yet. Antibody therapies are one of the most common forms of treatment for posterior segment diseases and were one of the earliest to reach the market. Several antibody therapies are FDA-approved for conditions such as wet AMD and diabetic macular edema and function by binding specific proteins that are upregulated in disease to prevent their normal function [10]. Finally, ocular prostheses are gaining interest as a method for sustained long-term therapeutic delivery and several devices, including intraocular lenses (IOLs), keratoprosthesis (Boston K-Pro), and the Argus II retinal prosthesis have already received approval for the market.

An important factor to consider for the safety and efficacy of ocular therapeutics is the route for delivering each of these treatment types into ocular tissues. Certain routes of delivery will elicit different responses and potential risks [7]. Each of these therapeutic modalities vary in immune and inflammatory responses, as well as the duration of delivery. Implants, nanoparticles, and viral therapeutic delivery systems each offer distinct advantages and challenges, making them valuable in advancing various forms of therapy, including gene, antibody-based, and cell therapy [9,11]. In this review, the most up-to-date ocular therapy modalities and delivery routes will be discussed and compared, along with other methods that show promise as future therapeutics. The advantages and disadvantages of each therapeutic system will also be described, along with current clinical trials and FDA approvals.

## 2. Types of Therapy

**Figure 1:**
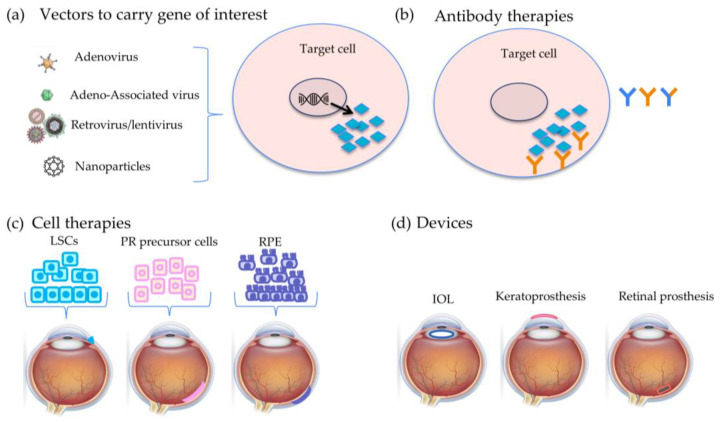
Overview of current ocular therapy types. There are several available and upcoming molecular and cell therapies for ocular diseases, including gene therapies, antibody therapies, and cell therapies, as well as devices. (**a**) Gene therapy involves delivery of a transgene of interest to the cell that then results in expression of a particular protein that is affected in a given disease. (**b**) Antibody therapies focus on binding to proteins that are overexpressed in the pathological state to attenuate disease. (**c**) Retinal pigment epithelium (RPE), limbal stem cell (LSC), and photoreceptor (PR) precursor therapies as cell sheets or suspended cells are being evaluated in late disease stages after advanced cell degeneration has progressed. (**d**) Prosthetic devices that are currently FDA-approved for the market are intraocular lenses (IOL—blue ellipse) to replace cataractous lenses, the keratoprosthesis (pink outline) for corneal grafts, and the retinal prosthesis (orange box) for late-stage retinitis pigmentosa (RP) patients. RPE, retinal pigment epithelium; PR, photoreceptor; LSC, limbal stem cells; IOL, intraocular lens.

Ocular therapies are used to treat a variety of diseases, ranging from anterior segment diseases, such as dry eye, cataracts, and glaucoma, to posterior segment diseases, such as RP and AMD (Figure 1). Molecular therapies are the primary choice to treat early- to mid-stage diseases and require some viable cells to still be present. Currently approved strategies that address the early stages of disease primarily utilize gene therapies, while single-target therapies and antibody therapies are approved for later-stage retinal diseases. Prosthetic devices that are currently approved include the intraocular lens (IOL) and the Boston keratoprosthesis (Boston K-Pro), used to treat anterior segment diseases, and the Argus II for treating retinal disease.

### 2.1. Gene Therapy

Gene therapy has been gaining a lot of attention in recent years for the treatment of ocular diseases with known genetic causes, especially retinal diseases. The retina is an ideal target for gene therapy delivery systems, since it is localized deep within the eye, making it less accessible for surface delivery. Moreover, its enclosed location and immune privileged status make it more amenable for testing gene therapies [9]. Gene therapy can halt or prevent disease and restore vision, provided that the affected cell type is still present. Several approaches to gene therapy have been developed based on the nature of various mutations that have been identified in retinal diseases, and these approaches are discussed below.

#### 2.1.1. Gene Augmentation

Gene augmentation, or gene replacement, is an approach that replaces a dysfunctional gene with a functional copy to promote the production of functional proteins [9,12]. This approach is simple and directly utilizes a viral or non-viral vector to deliver the functional gene. Gene replacement can be used in recessive inherited ocular diseases with monogenic mutations [9,12]. The advantage of this therapy approach is the ability to deliver the functional gene to specific cell types using target-specific vectors [9]. Some conditions such as RP have over 100 causative genes identified so far, and developing gene therapies for each gene is not practical, as the cost would be too great [13]. Moreover, the technique itself is limited by the carrying capacity of the vectors, with the most commonly used vector type being AAVs, which are limited to approximately 4.7 kb [14]. The high costs that are associated with treating a single patient is another disadvantage and can be anywhere between USD 400,000 and USD 2.1 million [15].

Despite some of the disadvantages, the therapeutic potential of gene augmentation has been demonstrated in the clinical setting with the therapy, Luxturna (voretigene neparvovec-ryzl), which is FDA-approved for pediatric patients with *RPE65*-associated Leber congenital amaurosis (LCA) and confirmed biallelic *RPE65*-mediated retinal dystrophy [16]. This gene therapy works by using a modified AAV2 vector to deliver the normal human *RPE65* gene subretinally to retinal cells, which restores vision. A phase III trial showed significant improvements from the baseline in terms of mean bilateral multi-luminance mobility test scores in the voretigene neparvovec treatment group compared with the control group at 1 year. The beneficial effects of Luxturna were maintained for up to 4 years of follow-up [17]. Notably, EIAV-ABCA4 (SAR422459), a recombinant lentiviral vector based on the equine infection anemia virus, has been developed for subretinal delivery of a copy of the normal coding sequence of the human *ABCA4* cDNA into the host genome. Studies in mice using this vector have shown cellular transduction in retinal pigment epithelium (RPE), photoreceptor (PR), and some other cells of the inner neural retina [18]. Recently, the three-year safety results of the EIAV-ABCA4 gene therapy trial in *ABCA4*-associated Stargardt disease patients showed that this therapy is relatively well tolerated [19].

Clinical trials are also ongoing for gene therapy treatments for rare genetic diseases. The two-year interim results of an ongoing Phase I/II gene augmentation therapy trial assessing the safety of the subretinal delivery of an AAV2 vector carrying human choroideremia (*CHM*)-encoding cDNA detected no systemic or vector-related toxicities. Furthermore, these results demonstrated that visual acuity was within 15 letters of baseline after the subfoveal AAV2-hCHM injections in 13 out of 15 CHM patients [20]. Several other gene augmentation therapies are in clinical trials for other inherited ocular diseases including achromatopsia, Leber hereditary optic neuropathy, RP, X-linked RP, X-linked retinoschisis, and Usher syndrome. A major downside to this approach is that it is not beneficial in diseases with an unknown genetic cause or mutations in more than one gene. 

#### 2.1.2. Modifier Gene Therapy

Similar to gene augmentation, modifier gene therapy involves the introduction of a single functional gene copy into targeted cell types; however, the advantage of this therapy compared to augmentation is that it can be used even when the underlying genetic mutations are unknown [9]. The modifier gene is not always the cause of disease and instead affects pathways that are upstream or downstream of the causative dysfunctional gene, altering the disease outcome [9]. Multiple pathways are perturbed in polygenic diseases, and recent work from our lab and others demonstrates that even in Mendelian disease, several other pathways are misregulated in addition to the primary mutation. Therefore, modifier gene therapy offers a broad-spectrum therapeutic that may be more effective than a single gene therapy, because it regulates multiple gene pathways that are impacted. Another advantage of this therapy compared to augmentation is that it can be used in Mendelian or complex ocular diseases if the dysfunctional genes are in pathways that are modulated by a modifier gene [9]. Modifier gene therapy is also advantageous in diseases with an unknown genetic cause if the affected pathways have been identified.

Many modifier genes are still under investigation and being identified, and polygenic diseases with mutations in genes in multiple pathways may not be affected by a single modifier gene. Modifiers mitigate the impact of the primary mutation on the rest of the gene networks that are affected in the presence of the primary mutation. The potential of this therapy is currently being evaluated in clinical trials. Our lab developed two modifier therapies using the nuclear hormone receptors (NHRs), *NR2E3* and *RORA*, and the clinical trials for these therapies are being spearheaded by Ocugen Inc. A phase I/II clinical trial using an AAV to deliver *NR2E3* subretinally in patients with *RHO-* and *NR2E3*-associated RP and *CEP290*-associated LCA has demonstrated both the safety and efficacy of *NR2E3* modifier gene therapy in multiple diseases [9] (NCT05203939). Two additional trials are also being performed using AAV to deliver *RORA* in patients with geographic atrophy secondary to dry AMD (NCT06018558) and Stargardt Disease (NCT05956626). An alternative to resetting the genes and pathways that are associated with retinal diseases may be targeting and potentially repurposing retinal cells through optogenetic therapy. 

#### 2.1.3. Optogenetics

Optogenetics is an approach that is used to activate certain non-light-sensitive neurons or other retinal cells by the expression of light-sensitive proteins called opsins within these cells [12]. Optogenetics has therapeutic potential for later stages of retinal diseases such as RP and Stargardt Disease after photoreceptor degeneration. Opsin genes are delivered to retinal cell types other than the photoreceptors, such as bipolar cells and retinal ganglion cells (RGCs), and subsequently activated with light [21]. Microbial opsins and animal opsins are the two types that are utilized in optogenetics, and they differ in their function, light sensitivity, and use for vision rescue [12,22]. Microbial opsins have a direct effect on ion channels and pumps through a conformational shift, whereas animal opsins indirectly affect ion channels via G-protein-coupled receptor (GPCR) signaling cascades during light absorption [23]. Different opsins can also elicit either “off” or “on” responses in the targeted cells, which is dependent on the desired effect of this therapeutic approach [24]. Preclinical gene therapy delivery of Channel rhodopsin-2 (*ChR-2*), a microbe-derived opsin, in *rd1* mice in both ON bipolar cells and RGCs led to increased cell responses in retinal explants in addition to improved visual responses in the cortex [25]. The downside to microbial opsins is their potential immune response to exogenous proteins compared to animal opsins. Utilizing *ChR-2* is also limited by light sensitivity, since the opsin is microbe-derived, and a behavioral study of treated animals demonstrated that intense blue light stimulus was required to elicit a response [25]. A phase I/II clinical trial using optogenetics in advanced RP is currently underway to evaluate the safety of this approach (NCT02556736). Patients received an intravitreal injection of AAV2 carrying *ChR-2* targeting RGCs and are being monitored for severe adverse events resulting from the therapy for up to 6 months after treatment. The efficacy of optogenetic therapy is unclear from preclinical studies and still needs further investigation. In cases where gene augmentation or modifier therapy may not be ideal, another therapeutic option that could improve the disease outcome by eliminating or editing the mutated gene is gene editing.

#### 2.1.4. Gene Editing

An approach that has gained much attention for gene therapy in recent years is gene editing with CRISPR/Cas9. Gene editing has the potential to alter the expression of or remove mutations from a specific gene and shows promise in dominant inheritance ocular diseases. The CRISPR/Cas9 system uses a guide RNA to direct an endonuclease (Cas9) to a specific sequence in a gene, where it causes a double-stranded break to allow for the modification of the genetic sequence [9,12,26]. Gene editing has similar advantages to gene augmentation, in that it is a directed approach that targets the causative gene in monogenic disease phenotypes [9]. A unique advantage of gene editing with CRISPR/Cas9 is that it provides a permanent and precise alteration to the genetic mutation [9,26]. However, there are several disadvantages to gene editing. The genetic mutation cannot be polygenic [9], and the guide RNA (gRNA) size is also limited to 17–24 bp [27]. Off-target mutagenesis effects occur at a frequency of over 50% with conventional CRISPR/Cas9 systems. More recent studies are trying to address this using Cas9 variants that minimize such effects, including the Cas9 nickase (Cas9n) variant and SpCas9-HF1, and also by improving the design of guide RNAs [28,29,30,31]. Another unwanted side effect is apoptosis, triggered by double-stranded breaks that are required for the CRISPR/Cas9 gene editing mechanism [32]. In light of these findings, a phase I/II clinical trial is underway to demonstrate the safety and efficacy of CRISPR/Cas9 gene therapy via subretinal delivery in patients with Leber Congenital Amaurosis Type 10 (LCA10), which is caused by the IVS26 mutation in the *CEP290* gene (NCT03872479). An alternate approach to reducing the expression of a mutant gene is gene silencing.

#### 2.1.5. Gene Silencing

Gene silencing is a potential therapeutic approach that utilizes small interfering RNA (siRNA) or microRNA (mRNA) to target and degrade the aberrant mRNA product of a dysfunctional gene, thus suppressing expression [9]. This approach shows potential in inherited diseases in which overexpression of a mutant gene is involved in the pathogenesis and uses natural means to reduce expression. Antisense oligonucleotides and siRNAs are among the most commonly used strategies for gene silencing [33]. The limitations of this therapy include the poor bioavailability of siRNA, RNA instability, potential degradation in the cells, and off-target effects due to non-specific targeting [9]. The half-life of unmodified naked siRNAs is a major challenge for the efficacy of gene silencing, given that they degrade within approximately 5 min in the bloodstream [34,35]. Taking into consideration the advantages and disadvantages, gene silencing is still a promising therapeutic approach that is currently in clinical trials for diseases such as LCA, RP, and Usher syndrome.

Currently, a phase II/III study is evaluating the safety and efficacy of intravitreal injection of sepofarsen, an RNA antisense oligonucleotide, in LCA patients with a specific *CEP290* mutation [36] (NCT03913143). Another active phase II/III clinical trial in patients with RP and Usher syndrome type 2 that possess mutations in exon 13 of *USH2A* is investigating the benefits of ultevursen, an RNA antisense oligonucleotide therapy (NCT05158296). Finally, there is a phase I/II clinical trial examining the effects of an RNA antisense oligonucleotide that targets mRNA from the *P23H* mutation in the *RHO* gene of RP patients (NCT04123626). The potential of gene silencing as a therapeutic for inherited retinal diseases is evident in the number of clinical trials that are currently taking place for various diseases; however, there are still some challenges to be further investigated to improve the effectiveness of this approach. One significant challenge is that this approach silences the gene with the mutation but does not provide a viable copy of the gene, which can be the focus of future studies. The challenge of extending the half-life of siRNAs is under investigation, and one study demonstrated that modifying siRNAs can extend their serum half-life to up to 72 h [37]. Another study showed that post-operative delivery of *Sparc* siRNA via nanoparticles in a mouse model of conjunctival scarring sustained gene downregulation up to 14 days after surgery [38]. This study shows the potential for reducing post-operative surgical failure after glaucoma filtration surgery or other ocular procedures, as well as for reducing the number of repeat injections that are needed. However, the sustained action of gene silencing is still very limited in comparison to the other forms of gene therapy and would likely require several repeat injections over short time frames to maintain efficacy. The limited duration also significantly impacts feasibility.

#### 2.1.6. Future Directions

Gene therapy as a whole is a promising and exciting therapeutic approach that has shown efficacy in several cases. One downside is that gene therapy is currently restricted to retinal diseases with mostly known genetic causes and can incur heavy costs per patient. Moreover, the majority of gene therapy strategies are viral and can trigger immunogenic responses and cause cell toxicity [39]. Gene therapy, however, is one of the treatment types for ocular disease that has FDA approval in one form of treatment, Luxturna, which is already on the market.

There are several forms of gene therapy that are currently under evaluation; however, each comes with its own advantages and disadvantages, which can inform the direction of future research. Future strategies for gene therapy need to consider the limitations of current technologies and address the following areas: accommodating large gene delivery and alternate delivery mechanisms that induce fewer immunogenic responses, such as non-viral methods of delivery, eye drops, and devices. Current research includes studying dual-vector therapies, in which a single gene is split into two transgenes with overlapping regions. The two transgenes are then delivered through two separate vectors and are joined via homologous recombination [40]. Other considerations for future directions include increasing the safety of gene therapies while reducing their toxicity. Gene editing and silencing also suffer from technological limitations, such as the size limit for gRNA in CRISPR/Cas9 systems and the short degradation time for siRNA, which should be addressed by future studies.

Current modifier gene therapies are setting the precedent for the effectiveness of broad-spectrum therapies, and future directions include using modifier therapy for disorders beyond the eye. Broad-spectrum modifier therapies that have been tested so far in the retina work by resetting multiple gene networks that are required for retinal function. This therapeutic option is well suited for complex diseases, such as AMD, that have multiple perturbed genetic pathways. Modifier gene therapy is also highly effective for single gene defects, where the primary mutation is the major driver of disease, and other gene networks impact the disease outcome [41]. The success of current modifier therapies in ocular diseases will pave the way for the use of modifier therapies for other ocular and non-ocular diseases.

Optogenetics is a potential gene therapy that is currently in clinical trials; however, there are serious gaps in knowledge that need to be addressed. Curiously, ChR-2 is a bacterial opsin and may not be the ideal choice of gene, since it is mainly responsive to blue light. Mammalian opsin genes, which confer responsiveness to a greater spectrum of light wavelengths, would likely be better suited for optogenetic therapy. Additionally, continued research expanding our understanding of how ChR-2 sends light signals to the visual cortex without photoreceptor machinery in bipolar cells and RGCs is critical for optimizing this therapeutic approach. Moreover, these cells lack the phototransduction machinery, including receptors that are required for the visual cycle. Certain RGC populations are intrinsically photo-sensitive; however, they function in circadian pathways and not in visual cortical pathways [42]. Thus, additional studies are needed to explain how these cell types can convert light into an electrical signal and propagate it to downstream neurons in the visual pathway. Studying the effect of *ChR-2* in bipolar cells and RGCs in the visual cortical pathway is also essential to demonstrate its efficacy as a therapy for retinal diseases. 

Further studies in the gene therapy space that could be carried out include testing combination therapies with both gene editing to remove the mutation and gene augmentation therapy to replace functional copies of the gene. Modifier therapies can also be tested in combination as a supplement to single gene augmentation therapies and may confer a more robust and sustained effect. Similarly, other future directions include combination therapies for diseases in which the patient suffers from more than one mutation. An alternative type of therapy that shows promise in ophthalmic diseases is antibody therapy.

### 2.2. Antibody Therapy

Antibody therapy is the use of antibodies that can bind and inhibit certain pathological molecules in ocular diseases to improve vision. Anti-VEGF therapy is the most widely used to treat wet AMD and is the first to be FDA-approved in the market. Monoclonal antibodies hold the potential to bind with high specificity to molecular targets that are involved in disease pathogenesis [43]. This therapy has also shown potential for treating ocular diseases that involve angiogenesis or inflammation and is primarily used for AMD and diabetic macular edema. AMD is a highly investigated disease for both single- and dual-target antibody therapies.

#### 2.2.1. Single-Target Therapies

Single-target therapies target one specific molecule that is involved in disease and bind to it. Anti-vascular endothelial growth factor (anti-VEGF) is the most well-known type of single-target therapy in ocular disease and is currently being investigated for wet AMD, diabetic macular edema, diabetic retinopathy, and macular edema due to retinal vein occlusion [10]. One of the main disadvantages of antibody therapies is that they address the symptom of disease, which is neovascularization, and not the underlying genetic mechanisms, unlike gene therapy. Furthermore, there is a need for frequent injections, which in itself can cause inflammation that could contribute to disease progression. In addition, while this therapy prevents the growth of new vessels, it does not prevent new vessels from forming. However, this treatment has demonstrated the ability to improve vision function, inhibit neovascularization, reduce vessel leakage, and provide better results than previous treatment methods [10,44,45], making this a current treatment of choice for neovascular retinal diseases. Various types of intravitreal anti-VEGF agents have been developed over the years to improve the treatment of disease, beginning with pegaptanib, an RNA aptamer, which was the first FDA-approved agent. This therapy was able to reduce vision loss, attenuating disease progression; however, it did not result in significant improvement in vision [46]. Other agents that have been approved since pegaptanib and achieved better improvement in vision include ranibizumab (Lucentis by Novartis), aflibercept (Eylea by Regeneron Pharmaceuticals), and brolucizumab (Beovu by Novartis). Several clinical trials are investigating the efficacy of these anti-VEGF agents in AMD, diabetic macular edema, and diabetic retinopathy (Table 1).

Another investigated target for single-target therapy is complement cascade targeted therapy in AMD and diabetic retinopathy. The complement system is suggested to be active in chronic inflammatory diseases and contributing to their pathology [10,47]. Components of the complement system have also been identified in AMD drusen and the vitreous of diabetic retinopathy eyes [48,49]. Current clinical trials using the complement cascade approach are investigating its effects in AMD and Stargardt disease (Table 1). Izervay, a PEGylated aptamer, is a recently FDA-approved treatment for geographic atrophy that is secondary to AMD, targeting complement C5 [50,51,52,53,54]. Syfovre, a PEGylated cyclic peptide that inhibits complement C3, is another FDA-approved treatment for geographic atrophy that is secondary to AMD [51,54]. However, one of the disadvantages with Izervay and Syfovre is that some patients developed choroidal neovascularization in response to both treatments [50,55]. Single-target therapies are also limited by the specificity of their target, since some diseases can arise from multiple perturbations.

#### 2.2.2. Dual-Target Therapies 

Dual-target therapies target two different molecular components that are involved in disease pathology, as opposed to single-target therapies, and this is advantageous for use in patients who have disruptions in multiple disease targets. In the eye, dual-target VEGF and angiopoiten-2 (Ang-2) therapies are under investigation as potential treatments that provide better improvement than standard anti-VEGF therapies. Ang-2 is associated with vascular remodeling and is elevated in wet AMD, diabetic retinopathy, and retinal vein occlusion [10]. Faricimab is a first-in-class dual-target VEGF and Ang-2 therapy that recently received FDA approval for the treatment of wet AMD and diabetic macular edema [10,56]. 

Dual- and single-target therapies have shown promising results as therapeutic approaches for vascular and inflammatory ocular diseases, although they do possess some limitations, including the need for frequent injections up to every 2–3 months and potential intraocular inflammation [10]. Researchers are working to reduce these limitations through different delivery systems such as implants, which could provide prolonged delivery of these agents into ocular tissues. 

#### 2.2.3. Future Directions

Future directions must address two major limitations, which are that antibody therapies do not address the cause of the disease as a whole but just a symptom, as well as the inflammatory responses that occur due to frequent injections. Future directions should include identifying causative genetic mutations. Developing therapies that either target causative disease genes or master regulators that can redirect the gene networks impacting the disease outcome may be more effective treatment options. Inhibiting targets that are further upstream in the disease pathway would allow for the prevention of disease and potential reversal of the disease phenotype, which is not possible with current antibody therapies.

Using implants for sustained release of both single- and dual-target therapies could reduce the need for frequent injections. The biggest challenge with sustained release implants is the appropriate mechanical function of the device, which must be addressed in future studies. The development of novel nanoparticles that are capable of sustained delivery of antibody therapies for several months would also reduce the need for frequent injections, thereby reducing inflammatory responses. Additionally, improving the ability of various nanoparticle formulations, as well as contact lenses, to deliver antibody therapies to posterior segment tissue would entirely remove the need for invasive methods of delivery, such as the intravitreal and subretinal methods that are commonly used to deliver antibody therapies. For treating ocular diseases in the advanced stages, cell therapies may be an effective option when most of the cells have degenerated.

### 2.3. Cell Therapy

Cell therapies are another promising therapeutic approach that involve using stem-like precursor cells and inducing differentiation of specific cell types that are affected in a particular eye disease and delivering or implanting the cells in the affected tissue area to improve vision. They can be used in cases in which the underlying genetic mutations are unknown and also in cases of very advanced disease stages with complete loss of the affected cell type. Cell-based therapies are currently in development for ocular disorders of both the anterior and posterior segments. The source of the stem-like cell population depends upon the target cell type. Cell therapies for corneal disease are the most commonly researched among anterior segment diseases, owing to the immune privilege status of the cornea, which would prevent the graft rejection immune response [57]. Corneal therapies are derived from limbal stem cells (LSCs) and mesenchymal stem cells (MSCs). The other ocular tissue for which cell therapies are being researched is the retina, with embryonic-like stem cells (ESCs) or induced pluripotent stem cells (iPSCs) and neural stem cells (NSCs) being used for the generation of RPE and retinal cell types.

#### 2.3.1. Limbal Stem Cells (LSCs)

LSCs are a population of stem cells that are located in the palisade of the Vogt region in the limbus and contribute to corneal cell replacement and regeneration after corneal injury. The unique regenerative capacity of LSCs, combined with their accessible location, makes LSCs a prime candidate for cell therapies to treat corneal injuries [57]. Corneal burns and injuries in the limbal region can lead to the loss of this stem cell population, resulting in conjunctival invasion of the cornea and neovascularization, reducing corneal transparency and leading to vision loss. Consequently, repopulation of the limbal region with LSC therapy is currently under study for the treatment of corneal disorders resulting in opacification [57]. LSCs for transplantation can be derived from a variety of tissue types, including the conjunctiva and the keratolimbal regions, and from either live donors or cadavers, while transplants can be derived from the patient, which are autologous transplants as opposed to allogenic transplants from a donor [57,58]. The most widely used method is the conjunctival–limbal autograft, owing to its success compared with other methods, and involves the transplantation of a piece of limbal tissue from the contralateral eye of the same patient [57]. The biggest disadvantage is that this procedure requires transplantation of up to one-third of the limbal tissue from the contralateral eye and could result in damage to the healthy eye [59,60]. A procedure that does not have this limitation is the culturing of stem cells using allografts from cadaveric donors, which reduces the chances of graft rejection and transmission of diseases such as melanoma from the donor [61,62]. This method has also been performed using autologous cultured limbal epithelial transplantation methods, in which limbus tissue samples are taken from the healthy eye of the patient and cultured separately, followed by the transplantation of the cultured cells in the limbus. This procedure led to promising results, and up to 76.6% of corneal chemical burn patients developed corneal transparency through this procedure [63]. A clinical trial for optimizing the process for manufacturing cultivated autologous LSCs is currently being developed, and if successfully applied in the clinic, this process could potentially be used to create effective, standardized treatments for corneal injury [64]. Current studies are also looking into the use of other stem cell types as an alternative to LSCs, due to the scarcity of LSCs.

#### 2.3.2. Mesenchymal Stem Cells (MSCs)

MSCs are a stromal cell population with the capacity for multi-lineage differentiation. Recent studies have examined MSCs as a potential alternate source of stem cells for LSC transplant therapy for corneal injury. MSCs that were derived from the bone marrow have been used successfully for corneal cell therapy in a previous clinical trial. This study showed a success rate between 76.5 and 85.5% for the MSC-derived cell therapy compared to a success rate of 72.7–77.8% for conjunctival–limbal autograft [65]. The study also showed a good safety profile for MSC-derived corneal cell therapy. Immature dental pulp stem cells, as well as stem cells that are derived from adipose tissue are also both being researched as a potential cell therapy for corneal damage [66,67]. The main advantage of adipose tissue-derived stem cells is their availability compared with bone marrow-derived stem cells. Furthermore, *in vitro* studies showed that adipose tissue-derived stem cells can attenuate the immunogenic response, which can speed up the process of corneal cell regeneration [68,69]. More preclinical and clinical studies testing adipose stem cell therapies for corneal diseases are needed, as they are an accessible alternative to bone marrow stem cell therapies for corneal injury. MSCs are mostly being tested for corneal grafts, and an alternative stem cell type is required for cell therapies to replace other cell types are human embryonic-like stem cells (hESCs), which are under evaluation for posterior segment diseases.

#### 2.3.3. Human Embryonic-like Stem Cells (hESCs)

The RPE is the most amenable to cell therapies among the tissues that are affected in retinal degenerative diseases, since RPE cells do not require the formation of synaptic connections, unlike neuronal cell types [57]. There are several clinical trials underway to test the use of hESC-derived RPE cell therapies, which includes a study showing a good safety profile without ocular inflammation, graft rejection, or tumor formation [70,71]. Furthermore, a recent clinical study carried out in a group of seven patients with Stargardt Disease, a maculopathy, showed safety over a 5-year period, and most of the patients showed increased or stable visual function [72,73]. Several clinical trials are currently underway for RPE cell replacement therapy for retinal diseases (Table 1). This includes a phase I/II trial testing human retinal progenitor cells in patients with RP, and early results showed improvements in the visual acuity in patients (NCT02464436). However, there are no FDA-approved treatments as of yet.

Neuronal cell types are some of the most challenging for designing cell-based therapies in the retina, since in addition to the issue of cell integration, neurons must also form synaptic connections with the appropriate neurons. Tests of cell therapies for RGC replacement in glaucoma and optic neuropathy are underway at the preclinical stages. The ethical implications of using ESCs has resulted in the testing of autologous stem cell-derived RGCs for use in cell therapies. However, autologous cell therapies cannot be mass produced, severely limiting their clinical application [57]. In addition, the same genetic causes resulting in RGC loss in the patient would be propagated with the use of autologous cell-derived therapies for RGC replacement [57]. Cell therapies for RGCs are made especially challenging because cell therapy-derived RGC axons must be able to innervate thalamic neurons [74]. The issue of RGC integration and synaptogenesis also remains a major obstacle. Recent preclinical studies are focusing on stimulating synaptogenesis by inducing ocular inflammation and also by knocking out certain transcription factors [75,76,77].

Photoreceptor cell therapy is another potential ESC-derived therapy for very advanced forms of retinal degeneration. Photoreceptor therapy has similar challenges to RGC therapy, including cell integration and synapse formation. Subretinal delivery of human photoreceptor precursor cells into dogs with inherited retinal degeneration resulted in differentiation of the precursor cells and integration into the surrounding tissue, and these grafts were active for 3–5 months, with the caveat that immunosuppression was also required for long-term graft survival [78,79]. Another study used ESCs to generate photoreceptor progenitor cells, induced using a recombinant retinal laminin isoform, which was then transplanted into the *rd10* mouse model of retinal degeneration. Results from the 20-week follow-up showed an absence of tumor growth, synaptogenesis, and improvement in visual function [80]. A clinical trial is currently underway for the use of retinal stem cells and progenitor cells for the treatment of AMD (NCT05187104).

ESCs are also used for anterior segment diseases, such as cataract formation. The most common treatment is replacement with a synthetic intraocular lens, which comes with the issues of insufficient power of the lens, calcification, and posterior capsular opacification [81,82]. This is especially problematic when the cataract is an inherited form of the disease and is present in infants. One study left the lens epithelial SCs intact while removing the remainder of the cataractous lens in infants, with results showing that the lens was rebuilt within 6 months [83]. One of the biggest hurdles with ESC-derived cell therapies are the ethical issues surrounding the use of ESCs in research and treatments, necessitating an alternative approach.

#### 2.3.4. Induced Pluripotent Stem Cells (iPSCs)

Cell therapies that are derived from iPSCs are an alternative to ESC-based therapies to replace cell types that have fully degenerated in diseases. The advantages of iPSC-derived cell therapies are that the source population, which are the iPSCs, can be taken from the patient, and some of the ethical considerations of hESCs do not apply to iPSCs. A study used iPSCs that were derived from an AMD patient to generate an RPE cell layer that was transplanted subretinally in the patient. The follow-up showed that while the RPE cell tissue layer remained intact, the best corrected visual acuity (BCVA) of the patient did not change, and macular edema was not resolved [84]. The absence of an improvement in visual function could likely be because the patient’s cells carried the mutations that resulted in the disease, which would also be propagated in the iPSCs that were derived from said patient. A better solution would likely be to obtain iPSCs that were derived from healthy donor tissue, ideally from close relatives, who have no co-morbidities or known diseases. Another disadvantage of using iPSCs is the reduced survival and integration of the cells into the ocular tissues. Further studies are needed to test the efficacy of iPSC-derived RPE cell therapy before being clinically approved. Perhaps, patient-derived iPSCs would be most useful to evaluate the effectiveness of therapies in a precision medicine manner. Similar to iPSCs, neural stem cells (NSCs) are an alternative cell population that can be used for differentiation of neuronal cell types.

#### 2.3.5. Neural Stem Cells (NSCs)

NSC-derived cell therapies have mainly been restricted to their neuroprotective effect on photoreceptors. NSC transplantation in cases of optic neuropathy due to elevated intraocular pressure resulted in the expression of the RGC marker, β-tubulin, and integration into the surrounding RGC tissue. However, these cells did not differentiate fully into RGCs, and the retinal function did not change with this treatment [85,86]. There is currently no evidence showing regeneration of RGC axons with NSC therapies [87]. On the other hand, NSC transplantation in the Royal College of Surgeons (RCS) rat model of retinal degeneration with dysfunctional RPE cells resulted in phagocytosis of photoreceptors’ outer segments by NSCs, demonstrating the neuroprotective effect of NSCs on photoreceptors [88]. However, transplanted NSCs do not differentiate into photoreceptor cell types and serve a neuroprotective function rather than function as a cell replacement therapy [89]. Overall, future research can focus on developing stem cell therapies that are capable of fully differentiating into specific cell types and can integrate into the surrounding tissue, as well as form the appropriate synaptic connections in neuronal cell types. 

#### 2.3.6. Future Directions

The biggest challenges that need to be addressed for cell therapies in future research are the integration into the surrounding tissue and the differentiation and normal function of the cell type that the therapy must replace. LSCs are the most successful form of cell therapy thus far for corneal diseases, as these cells can be derived from healthy donor limbal tissue or unaffected patient tissue. The next steps for LSC therapy should focus on improving the feasibility of using cultured LSCs for corneal grafts. Current work is already underway for optimizing a manufacturing protocol for large-scale generation of LSCs [64], and future studies can work on implementing this protocol into clinical practice.

Cell therapies are particularly challenging for the replacement of posterior segment tissue, including both RPE and neuronal cell types. Further research on RPE cell therapies can differentiate iPSCs that are derived from normal patients into RPE cell types and test this as a form of cell therapy in patients with RPE diseases, such as RP and AMD. A major hurdle to overcome in neuronal cell replacement therapies is to improve the integration of photoreceptors and RGCs into existing circuitries of the retinal and visual pathways. In addition, future directions should aim to achieve complete differentiation of stem cell types, such as ESCs, iPSCs, and NSCs, into fully functional retinal cell types. An alternative approach that would avoid some of the challenges of cell therapies would be prosthetic devices.

### 2.4. Prostheses

Prosthetic devices are designed to replace ocular structures and specific cell types within the structures in cases of complete cell loss, similar to cell therapies. There are three main types of prosthetic devices that are either approved or being tested for eye diseases, and these are retinal prostheses, intraocular lenses (IOLs), and keratoprostheses. IOLs have seen major success as an FDA-approved device with many options available on the market, and keratoprostheses are successful in alleviating complications associated with corneal graft failure. Retinal prostheses have also seen recent success in the form of FDA approval and are continually being developed and evaluated.

#### 2.4.1. Retinal Prosthesis

Retinal prostheses are a novel treatment approach for advanced forms of ocular disease, in which the affected cell type has fully or almost fully degenerated, and where gene and cell therapies would not be a viable option. Retinal prostheses are devices composed of a series of small light-sensitive photodiodes that convert light to electricity [90]. This has potential applications in retinal diseases with photoreceptor degeneration, such as RP, by converting light to electrical impulses that can be transmitted to retinal cell types downstream of photoreceptors, such as bipolar cells [90]. The epiretinal prosthesis is one type of retinal prosthetic consisting of an external photo-sensing camera that converts light into an electrical signal that gets transmitted inside of the eye to the stimulator chip containing small circuitry that streamlines the signal to the electrodes facing the retina, transmitting the electrical signal to these neurons [90]. Some of the challenges to be surmounted with retinal prostheses include determining whether the inner retinal neurons are viable and able to transmit signals to downstream visual pathways, the stimulation threshold for these neurons, and their ability to translate these signals into visual inputs [90]. Inner retinal neurons such as bipolar cells and RGCs may not be viable at much later stages of diseases such as RP. The stimulus threshold for inner retinal neurons is typically higher than for outer retinal neurons such as photoreceptors, since photoreceptors amplify the signals under normal physiological conditions. 

Pilot testing in humans suggested that individuals with advanced retinal degeneration could perceive spots of light with electrical stimulation of handheld electrodes placed in the intravitreal space [91]. However, further testing with epiretinal electrode arrays showed inconsistent results. Stimulation of a single electrode did not always result in perception of only a single shape, and stimulation of multiple electrodes sometimes only led to perception of a single shape [90,92]. Currently, there are approved retinal prostheses that can be implanted in patients with photoreceptor degenerative diseases such as RP and AMD. These devices include the Argus II epiretinal retinal prosthesis system, the subretinal Artificial Silicon Retina (ASR), and the PRIMA subretinal. The subretinal Alpha-IMS is CE-approved according to the standards set by the European Medical Agency (EMA). The Argus II Retinal Prosthesis System was FDA-approved in 2013 for the treatment of advanced RP in patients [93]. It was discontinued in 2019, with plans to replace this device with the ORION cortical device, which is designed to transmit visual information directly to a chip that is implanted in the cortex and is still under development [94]. Additional disadvantages of prostheses include the specificity of patient qualification and post-operative rehabilitation, which must be overcome in order for more patients to benefit from this treatment.

#### 2.4.2. Lens Prosthesis

Prostheses for anterior segment tissue have also been developed for regions including the cornea and lens [95,96] and are used in clinical practice. Cataracts are one of the leading causes of reversible blindness worldwide [97]. Synthetic intraocular lenses (IOLs) are the method of choice for replacement of cataractous lenses [95,96]. Lens prostheses have had a long history of success, since the lens is a transparent structure that provides refraction, a property than can be more easily recapitulated by a device than the electrochemical impulses that are necessary for the function of retinal implants. Current IOLs are typically manufactured as a single piece with two loops at each end for surgical incorporation into the eye and have a diameter of 6.0 mm. IOLs are made either from acrylic material, such as poly (methyl methacrylate) (PMMA), or silicone [95]. The procedure involves the removal of a portion of the anterior capsule, while leaving the remaining part of the anterior capsule and the full posterior capsule intact, generating a structure resembling a bag into which the IOL can be inserted [95]. The greatest advantage of a synthetic IOL is that donor tissue is not required for replacement and that it can potentially last throughout a patient’s lifetime. In addition, IOLs can also correct for existing visual abnormalities such as astigmatism [98].

One possible post-operative complication is posterior capsular opacification (PCO), which is cloudiness of the posterior lens epithelium resulting from the transition of the epithelial cell type to a myofibroblast cell type [99]. Recent advancements include the development of open-bag IOLs that keep the lens bag open post-surgery to allow for aqueous humor to bathe the tissue, reducing the incidence of PCO formation. One such device on the market is the Zephyr, a monofocal IOL that is shaped similarly to a wheel, with perforations along the outer edge that allows for a greater flow of aqueous humor. Another challenge with synthetic IOLs is accommodation. A normal human lens becomes more spherical in response to ciliary muscle contraction, allowing for clearer vision of nearby objects, while the lens becomes more elongated in response to ciliary muscle relaxation, allowing for clear vision of objects that are further away [95]. Several types of IOLs have been developed for clear vision of both nearby and distant objects. This includes multifocal lenses consisting of two or three sections with differing refractive power, extended depth of focus (EDOF) IOLs with an extended range of clear vision, as well as the accommodating IOLs that allow for a change in their shape in response to attempts by the patient to accommodate objects at different distances [95]. EDOF IOLs that are currently on the market include the Tecnis Eyhance by Johnson & Johnson, as well as the AcrySof IQ Vivity by Alcon Laboratories, while Crystalens (Bausch & Lomb Incorporated) is an accommodating IOL that is currently FDA-approved for the market. The development of the synthetic IOL has had a profound impact on treatments for ocular disease, as it can treat one of the leading causes of blindness worldwide. The cornea is another tissue that is the primary source of diffraction of light, and prostheses have also been developed for improving current corneal graft procedures.

#### 2.4.3. Keratoprosthesis

The keratoprosthesis is an anterior segment prosthetic device that is currently in clinical use and was developed as a result of corneal graft failure arising from complications of conventional corneal transplants, which is the most common method for treating corneal opacification [100,101]. The most commonly used keratoprosthesis is a type of core skirt keratoprosthesis, which is the Boston K-Pro prosthesis, consisting of an anterior plate composed of PMMA with an optical stem and a back plate made of titanium [102]. The donor corneal graft is placed between the two plates, and the entire prosthesis is attached to the host tissue. The biggest advantage of the use of keratoprosthetic devices is that it greatly reduces the chance of corneal graft failure in high-risk patients, including those with neovascularization and severe corneal burns, as well as patients receiving more than one corneal transplant [103]. The back plate containing holes also allows for an improved flow of aqueous humor through the corneal tissue, further reducing the risk of corneal graft failure [103]. 

Challenges that are encountered with the keratoprosthesis device include bio-integration and infections [102]. Poor bio-integration of the keratoprosthesis occurs due to low adhesion between the PMMA material and the host corneal tissue, which could potentially result in detachment of the prosthesis and leakage of aqueous humor [100]. The hydrophobic PMMA material often requires modification of its surface or wetting of the surface to improve cell adhesion. A previous study coated dopamine-activated PMMA with Calcium Phospate (CaP), which had better bonding to collagen hydrogel than uncoated PMMA [100]. However, the poor binding between the CaP and the dopamine-activated surface led to delamination between these components over time. A novel dip-coating method in which chloroform was used to create craters in the PMMA surface was developed, which allowed for greater retention of hydroxyapatite nanoparticles containing CaP. Corneal stroma fibroblasts showed greater bio-integration with and adhesion to the resulting surface-modified PMMA [100].

The second major challenge with keratoprosthesis is the development of infections with the surgical procedure. The current course of action in clinical practice is the use of prophylactic eye drops containing antibiotics, including Vancomycin, and antifungal medication such as Amphotericin B to prevent infections after surgery [104]. Research is currently underway to develop implants that are able to release the medication over sustained periods of time using animal studies [101]. A previous study incorporated Vancomycin into a hydrogel implant for potential use as part of a keratoprosthesis for sustained release of prophylactic medication for up to 7 days [101]. Testing in a rabbit model of corneal infectious keratitis showed a reduction in the incidence of *Staphylococcus aureus* infection in the group receiving a Vancomycin-treated hydrogel compared with the vehicle hydrogel group [101]. The keratoprosthesis has seen great strides since its development, and research is ongoing to improve its design and reduce the risk of post-surgical complications associated with its use.

#### 2.4.4. Future Directions

Future directions for retinal prosthesis should further study the mechanism of how light gets converted by the implant into an electrical signal, and how this signal is propagated along the visual pathway. This would help increase the sensitivity of existing retinal prosthetic devices, since the signal detection threshold for inner retinal neurons such as bipolar cells and RGCs are lower than photoreceptors [90]. Retinal prosthetic chips that are capable of converting information on wavelengths also need to be developed for accurate vision. Furthermore, additional work is required in standardizing the measurement of light perception to more accurately assess the efficacy of these implants.

Future directions in IOLs include improving the design of accommodating IOLs to function as close to the human lens as possible, as well as the development of IOLs that are capable of releasing drugs that may alleviate post-surgical complications such as PCO. Similarly, further work on keratoprosthesis includes continuing to develop novel nanoparticles and drug delivery systems to prevent infections. The development of a keratoprosthetic device using biosimilar a material that is more physiologically compatible with the corneal extracellular matrix may also reduce inflammatory responses and infections. Future work also needs to test these nanoparticle drug delivery systems being incorporated into both IOLs and keratoprostheses in human clinical trials for both their safety and efficacy in reducing surgical complications. 

## 3. Mode of Delivery

Understanding the current types of therapies that are available and under investigation for ocular diseases is paramount for the advancement of therapeutics and improvement in patient outcomes; however, the method for delivering these therapies into the eye and ocular tissues must also be considered. Many of the delivery methods currently used for ocular therapeutics are invasive, expensive, and/or lack sustained action. Injections are the most common delivery mode utilized for the delivery of gene, cell, and antibody therapies to the posterior segment tissues. Additionally, some therapeutics including gene therapy also require a vector, either viral including adeno-associated viruses or non-viral such as nanoparticles, to deliver them into targeted cells within the ocular tissue. Therapies for anterior segment conditions can be delivered via injections, eye drops, or implants. Furthermore, drug-eluting contact lenses are a delivery mode that has potential for use in ocular therapies. The advantages and disadvantages of these delivery modes will be discussed in the following section, along with future directions.

### 3.1. Injection Routes

**Figure 2:**
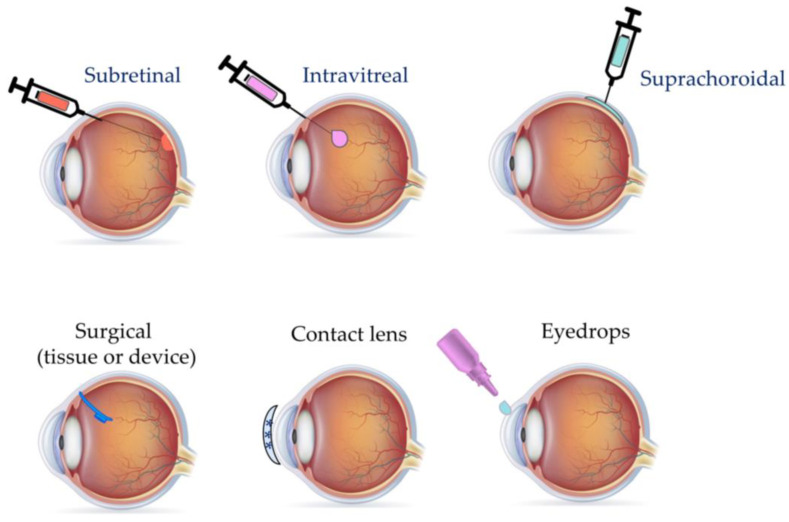
Routes of ocular therapy delivery. The most commonly used modes of delivery of therapies for anterior segment diseases are eye drops and contact lenses loaded with therapies such as drugs. Posterior segment disease therapies are typically delivered through the intravitreal, subretinal, and suprachoroidal routes.

The retina can be accessed through three different routes for injection of therapeutics, subretinal, intravitreal, and suprachoroidal [7], with intravitreal and subretinal being the most commonly used (Figure 2). The delivery of viral and non-viral vectors through these routes has distinct advantages and challenges. Furthermore, the choice of which route to use depends on the cell type that is targeted.

#### 3.1.1. Intravitreal 

Intravitreal delivery involves placing therapeutics or devices directly into the vitreous cavity, which contains the vitreous humor. Presently, intravitreal injections are used for delivering medications such as antibiotics, steroids, and antibody therapies, including anti-angiogenic therapies for AMD [105], and diabetic retinopathy [106], as well as for placing implants that slowly elute drugs. This delivery route is also commonly used for delivering experimental therapeutics for glaucoma to RGCs. Intravitreal injections are relatively safe, less invasive, and simple procedures, since they may be performed in the doctor’s office [107]. The tradeoffs of this route are that it is considered less invasive than subretinal injections and that it is potentially capable of accessing the entire inner retinal surface, but at the cost of dilution of the therapeutic in the vitreous fluid and immune responses reducing efficacy. Another limitation of intravitreal delivery is limited vector transduction into outer retinal cells due to the barrier of the inner limiting membrane and the vitreous humor [108]. These limitations may be overcome through modifying the delivery vehicle [109] or momentarily disrupting the barrier to induce effective transduction of the outer retina [108]. Another disadvantage of this delivery route is that it often induces a strong humoral response that can lead to inflammation or elimination of the treatment, thus reducing efficacy [7]. Some of the other disadvantages of intravitreal delivery are increased intraocular pressure, as well as dilution of therapeutics, such as drugs, and gene and antibody therapies, within the vitreous body [110]. Subretinal delivery is another approach that can be used for therapy delivery when the disadvantages of intravitreal, such as targeting the outer retinal cells, impede efficacy.

#### 3.1.2. Subretinal 

The subretinal route is used to more effectively treat conditions affecting the photoreceptor (PR) layer and the RPE. This route is used in therapies for several retinal diseases, including gene, antibody, and cell therapies for both exudative and non-exudative AMD, diabetic retinopathy, and Stargardt AMD. There are three major approaches for subretinal injections. The first is the transcorneal route through the cornea, past the lens, and through the retina [111]. The second is the trans-scleral route through the sclera, choroid, retina, and the vitreous into the subretinal space, which is a commonly used method in clinical and preclinical studies [112,113]. This route is used for the subretinal delivery of Luxturna. The last method is the least invasive, which is the external trans-scleral route through the sclera and choroid into the subretinal space [114]. There are several advantages to the use of subretinal injections for diseases affecting the outer retina and RPE. Gaining access to the subretinal space (SRS) facilitates direct contact of the drug with the PR and RPE layers, which optimizes the drug concentration in these cells. The SRS is a safer location than the intravitreal space due to its immune-privileged status and closed area. Furthermore, subretinal injections require lower doses of therapeutic drugs [110]. The major barrier to subretinal delivery is the blood–retinal barrier, formed by the retinal vasculature, which limits the types of therapies that can be delivered using this approach to those that can be taken up by retinal or vascular endothelial cells, such as gene and antibody therapies [115]. Permanent retinal detachment is an important risk and disadvantage of using this delivery route, since an iatrogenic retinal detachment is created after accessing the SRS [116], although iatrogenic retinal detachment usually heals and disappears within a week after the procedure [110,117]. Another disadvantage of subretinal injections is that they are an invasive procedure that only affects a localized region of the retina. However, this approach has minimal trauma, with rapid recovery of retinal structure and function if this procedure is performed with care by trained personnel [117]. If targeting a different region than the retina, then suprachoroidal delivery may be another route to utilize.

#### 3.1.3. Suprachoroidal

Suprachoroidal injections are used to deliver therapies for diseases afflicting the RPE and choroid, such as AMD and Stargardt Disease. Suprachoroidal delivery is a relatively new advancement and is less invasive than subretinal delivery. The suprachoroidal space (SCS) is found between the sclera and the choroid, which is usually mostly collapsed because of the intraocular pressure and the fibers that attach the sclera to the choroid [118]. The suprachoroidal delivery route provides a new and useful method for ocular therapy delivery, particularly in human patients, since it can minimize damage to the retina. The SCS is an appealing site of drug delivery because of its proximity to the choroid, a target for exudative AMD therapies, and because it is also proximal to the ciliary body, a target for anti-glaucoma treatments [119,120]. A lower drug dose can also be used to achieve the same efficacy as traditional routes of administration [120]. This method has shown promising results in large animal models and has been shown to be a safe approach in a clinical trial treating uveitis with macular edema [121]. However, accessing the SCS poses a disadvantage of suprachoroidal hemorrhage, so this procedure must be performed with this risk in mind. Similarly to the subretinal route, this route provides a localized delivery of the therapy to a portion of the SCS. This method requires specialized needles and may be difficult to carry out in smaller animals. In addition, the outer blood–retinal barrier consisting of the RPE poses a potential barrier for therapeutic delivery to photoreceptors. Potential immune reactions by macrophages in response to the therapy could be another barrier to suprachoroidal delivery [122]. Suprachoroidal delivery, along with the other routes, all possess unique tradeoffs that are dependent on the overall result that is sought and can still be improved upon.

#### 3.1.4. Future Directions

Intravitreal, subretinal, and suprachoroidal delivery are all considered invasive to varying degrees. Future research and therapeutic development should consider less invasive routes of delivery, such as topical or oral, and ways to improve the sustained action of therapies in order to reduce the need for multiple injections. Eye drops and contact lenses are topical delivery routes that are already utilized for some ocular conditions and are discussed below. Intravitreal delivery faces the challenges of reduced efficacy from dilution of the therapy and strong immune responses. Additionally, this route is less effective at transducing cells in the outer retina. Research should investigate the optimal dosage to maintain efficacy within the vitreous without inducing toxicity, as well as utilizing vectors or drugs that do not promote or even reduce the immune response. Using steroids in combination with the therapies is a typical recommended course. The main challenges of subretinal delivery that need to be addressed are the risk of retinal detachment and localized, not widespread, delivery. One solution could be that injections are given in multiple locations across the SRS to mitigate the limited area of effect of this delivery route; however, this would be at the cost of multiple invasive injections and increase the risk of retinal detachment. Further research should be carried out on ways to increase the area of effect for one subretinal injection. Suprachoroidal delivery faces similar challenges to subretinal delivery, with a risk of suprachoroidal hemorrhage and localized delivery into the SCS. Similar to future directions for subretinal delivery, ways to mitigate the risk of suprachoroidal hemorrhage or increase the area that is affected by one injection should be investigated. Solutions should include the discovery of vectors that can be used for carrying ocular therapies without the challenges of sustained delivery, improved transduction, and reduced immune response.

### 3.2. Vectors

Among the delivery methods, viral-mediated delivery has the highest risk of immunogenic responses that can lead to toxicity, but they also provide greater sustained action of therapeutics over several months compared to other types of vectors. Viral vectors can be classified into AAVs, Adenoviruses (Ad), and lentiviral vectors. Ad viruses were some of the earliest studied viruses for use in gene therapies; however, they are limited by the widespread prevalence of antibodies against Ad serotypes in the human population. On the other hand, lentiviral vectors, while higher in efficacy, have a greater risk of toxicity due to being derived from viruses that can integrate into the host genome, such as HIV-1. Consequently, AAVs are emerging as the most widely used viral vector for use in ocular gene therapy due to their higher efficacy and increased safety profiles when compared with the other two categories of viral vectors (Table 2) [123,124].

#### 3.2.1. Adenoviral (Ad) Vectors

Ad vectors are the most commonly used vectors for cancer cell gene therapy and are non-enveloped viruses containing double-stranded DNA that can effectively transduce dividing and non-dividing cells. Adenoviruses are an attractive vehicle for gene therapy due to their minimal risk of insertional mutagenesis [144] and packaging capacity of up to 37 kb [17]. Early studies identified the potential applications of Ad viral vectors for use in gene therapy for retinal disease [145]. The beta subunit of the cGMP phosphodiesterase gene (PDE) was delivered using an Ad viral vector in a mouse model of retinal degeneration, which showed delayed apoptosis of photoreceptor cells at 6 weeks post-treatment [145]. Another study performed in a mouse model of retinoblastoma showed that Ad-mediated delivery of the retinoblastoma tumor suppressor gene (Rb) reduced cell proliferation and suppressed the formation of pituitary melanotroph tumors [146]. Currently, VCN-01, an oncolytic vector based on Ad5, is an Ad vector in a phase I clinical trial of advanced solid tumors [147]. The goal of the study is to use VCN-01 to cause tumoral spread of this viral vector and increase the leakage of immune factors from blood vessels into the tumor environment, with the phase I trial showing safety [147]. However, there is a widespread presence of serotypes for Ad viruses in the human population, resulting in an increased number of neutralizing antibodies targeting this virus [148]. Thus, Ad vectors are not as widely used in ocular gene therapy clinical trials as AAVs due to the strong immune response that triggers inflammation and the elimination of the transduced cells [149].

#### 3.2.2. Adeno-Associated Viral Vectors (AAVs)

The most frequently used vectors for ocular gene therapy are AAV vectors, as they generate a lower immunogenic response compared with Ad viruses. This vector system is an ideal vehicle for human gene delivery [150]. At only 25 nm in diameter, AAV is one of the smallest viruses identified, and it is replication-defective, non-enveloped, and contains single-stranded DNA. Furthermore, the AAV vector system has several naturally occurring serotypes that present selective tropisms for different target cells in the retina [151]. Several of these serotypes can transduce genes into specific cell types which organize the neural retina through different delivery methods. Notably, intravitreal injection of AAV2 transduces RGCs [152], and subretinal delivery of AAV8 transduces photoreceptor cells [153].

Vectors that contain suitable cell specificity are necessary for gene therapy to be successful. AAV serotypes 1, 2, 5, 8, and 9 have shown the ability to transduce the RPE and/or photoreceptors in retinas of wild-type animals through subretinal injection [154], and high levels of retinal gene transfer have been attained using AAV serotypes 1, 2, 4, and 5 [125]. AAV2 is the most commonly used serotype, based on the effective transduction of retinal neurons and successful transduction of injured and diabetic eyes, better safety compared to lentiviral vectors, and lasting transgene expression [155]. Different AAV2 serotypes behave differently in the human retina. It has been determined that AAV2/4 and AAV2/5 are especially effective at transducing photoreceptor cells in the human retina, while AAV2/8 showed low transduction of photoreceptors, and AAV2/5 is very specific to the outer nuclear layer [126]. AAV5 transduces both RPE and photoreceptor cell types and shows long-term expression, as well as lower immunogenic responses compared with commonly used AAVs such as AAV2 [129,156,157,158]. Gene therapy for *RPE65* using AAVs has been shown to restore vision in a canine model of LCA [159]. AAV2 is the serotype that is used in Luxturna, the first FDA-approved directly administered ocular gene therapy [16]. Luxturna is delivered via subretinal delivery, and currently, only one injection is necessary to provide improvement for up to 4 years. Studies are ongoing to examine the longer-term efficacy of Luxturna and whether additional injections may be required. One disadvantage to using AAV vectors for gene therapy is that the carrying capacity of the vector is limited to around 4.7 kb [14]. Additionally, while AAVs elicit a lower immune response than other viral vectors, they do still cause it, which is another disadvantage. However, the AAV is the most successful viral vector that has FDA-approved treatments associated with it. For therapies requiring a larger carrying capacity than the limited capacity of AAVs, lentiviral vectors may be an alternative.

#### 3.2.3. Lentiviral Vectors

Lentiviruses are retroviruses that have an RNA genome and an ability to infect non-dividing cells due to the transport of their viral pre-integration complex into the host cell nucleus after infection [160]. There are five lentiviral serogroups: human immunodeficient virus 1 (HIV-1), equine infectious anemia virus (EIAV), simian immunodeficiency virus (SIV), feline immunodeficiency virus (FIV), and bovine immunodeficiency virus (BIV) [161]. Researchers have used lentiviral vectors containing a copy of the human *RPE65* gene delivered into *Rpe65*-deficient mice to restore cone function [162]. Additionally, the delivery of the wild-type human *ABCA4* gene into *Abca4*^−/−^ mice via lentiviral vectors was shown to correct the Stargardt Disease phenotype by reducing the accumulation of the lipofuscin pigment A2E [18]. Moreover, lentiviruses provide stable expression because of genome integration, have an extensive tropism, and have a broad capacity for cloning. Lentiviral vectors also have a larger carrying capacity, up to 10 kb, compared to AAV [163]. However, there are risks when considering lentiviruses as an ocular therapy vector system. There is a potential for insertional mutagenesis associated with retroviruses’ ability to integrate into the genome [164], and there is also a possibility that the lentiviral vector may produce replication-competent retroviruses within host cells [165], making them a less commonly utilized option for ocular therapies. One of the main disadvantages of viral vectors such as lentiviruses is the immunogenic response. In contrast, non-viral vectors such as naked plasmid do not elicit an immune response.

#### 3.2.4. Naked Plasmid

Clinical-grade plasmid DNA is prepared for the purpose of transferring a gene of interest into a tissue. The pEYS606 product is currently being evaluated at a preclinical level for the treatment of non-infectious uveitis [166]. This therapeutic product is a form of plasmid DNA containing the extracellular domain of the p55 TNFα receptor gene that has a high binding affinity to TNFα, fused to the human IgG1 Fc domain. The fusion protein gene was transfected into the ciliary muscle region in a rat model of uveitis using electroporation. The results showed reduced inflammation, a neuroprotective effect on photoreceptors, and efficacy at low concentrations [166]. This study led to the first phase I/II clinical trial testing the safety and efficacy of the pEYES606 treatment for uveitis (NCT03308045). Naked DNA is an appealing non-viral vector due to its inherent simplicity, ease of production in bacteria, and manipulation through standard recombinant DNA methods. It exhibits minimal spread and transfection to remote locations after delivery, which allows for multiple administrations in mammals without triggering an antibody response against itself [167,168]. One of the downsides to naked plasmid therapies is that they cannot produce more copies of the transgene of interest, unlike AAVs, and thus, they may have a less sustained effect, requiring multiple injections. Currently, there are no FDA-approved treatments using naked plasmid DNA; however, this would be an effective option for larger genes that exceed the carrying capacity of viral vectors if the sustained action could be improved in future research.

#### 3.2.5. Future Directions

While viral vectors are able to prolong the sustained action of therapies and target specific cell types, the main challenge of immune responses still remains. Future studies should focus on modifying viral vectors to eliminate immune response or find alternative vectors that do not elicit an immune response, while maintaining sustained delivery. Studies are ongoing for testing the duration of effect of the currently approved gene therapy, Luxturna, which is important for determining whether repeat injections are required, since frequent injections may increase inflammation and immune responses. Studies should also investigate modifying AAVs to improve their carrying capacity for conditions associated with large genes. Naked plasmid vectors pose a good alternative, given that they do not elicit an immune response and have a large carrying capacity; however, the issue of sustained delivery must be addressed to make them effective long-term therapeutic vectors. Finding less invasive ways to deliver vectors and therapies into ocular tissue, such as drug-eluting implants, eye drops, and drug-eluting contact lenses, could help reduce immune responses.

### 3.3. Eye Drops

Topical application of eye drops is the most commonly used treatment for ocular diseases of the anterior segment and is primarily used for the treatment of ocular surface diseases, such as dry eye disease, as well as for glaucoma [169]. The ease of use and minor side effects are some of the advantages, while some of the disadvantages include the need for frequent application due to leakiness through the nasolacrimal system, the lack of a sustained effect over a long duration, and low patient compliance [169]. Gel-based formulations can solve the problem of leakiness; however, gel eye drops can also result in temporary blurry vision and solidification of the formulation at the eyelid interface. In situ gelling systems have therefore been developed that are applied topically in liquid form and turn into a gel form upon contact with the ocular surface in response to a specific temperature, pH, or ion [170,171,172].

Most of the current treatments for ocular surface diseases are eye drop formulations, including Miebo, Restasis, a cyclosporin drug, and Eysuvis, an ophthalmic suspension with loteprednol etabonate, all of which are FDA-approved treatments for dry eye disease [173,174,175,176]. Approved eye drops for use in glaucoma include prostaglandins such as latanoprost, travoprost, tafluprost, and bimatoprost, as well as Rho kinase inhibitors such as Netarsudil [177,178]. Additional glaucoma treatments are cholinergic agonists such as pilocarpine; alpha-adrenergic agonists including apraclonidine and brimonidine; beta blockers such as timolol; and carbonic anhydrase inhibitors such as dorzolamide [179,180,181]. The main ocular barriers for uptake of eye drops by the iridocorneal angle tissue affected in glaucoma are the cornea, sclera, and conjunctiva, which are also the main routes of entry into the eye. The range of treatment options for dry eye and glaucoma demonstrate the efficacy of eye drops for anterior segment tissues; however, some limitations still need further investigation.

#### Future Directions

Eye drops are an accessible treatment modality that can be self-administered by the patient. They are versatile in treating a range of diseases, from corneal diseases, such as dry eye and infections, to glaucoma and inflammation. Increased drainage through the lacrimal system necessitates a higher frequency of administration than other treatment modalities, such as gene and antibody therapies. Future studies should examine the synthesis of novel eye drop formulations that are viscous, such as tear gels, to increase the ocular surface residence time while preserving normal optical clarity.

Barriers to uptake of eye drops into ocular tissues deeper than the cornea are another disadvantage that must be addressed in future studies. Glaucoma eye drops must pass through the cornea and sclera to reach the target iridocorneal angle tissue [182], and future studies could develop eye drop formulations to increase uptake. In addition, eye drops also do not readily cross ocular barriers to the posterior segment tissues, including the cornea, lens, and vitreous humor, and would diffuse in the vitreous. Less than 5% of eye drops reach the posterior segment [183]. An advantage of diffusion would be increasing the target area, while a disadvantage would be the loss of concentration, thus potentially reducing efficacy. Studies are currently being carried out to research drug formulations and methods that can more effectively deliver therapeutics to the posterior segment for retinal diseases. Some of the delivery methods under study for use in eye drop formulations for retinal disease are nanoparticles, which increase the ocular residence time of the drug and allow for deeper ocular penetration to posterior segment tissues.

### 3.4. Nanoparticles

Nanoparticles offer a promising approach to ocular delivery for not only drug therapies but also other types, such as gene therapies. Nanoparticles for ocular therapy use various chemical and physical techniques to deliver therapeutics into cells. Nanoparticles are generally engulfed by the target cells through endocytosis or phagocytosis. Within the eye, photoreceptors are mainly endocytic [184], while cells in the RPE show both endocytic and phagocytic properties [185,186]. The uptake of nanoparticles by their target cells also depends on their composition and charge. Nanomicelles, lipid-based liposomes, polymer-based nanospheres and dendrimers, and peptide-based nanoparticles are the main types of nanoparticles that are being tested for ocular delivery [187]. Nanoparticles are specially synthesized using various nanomaterials to achieve specific effects such as gene therapy delivery or sustained effects.

#### 3.4.1. Nanomaterials

Nanomaterials are the chemical compounds that serve as the starting materials for the synthesis of various nanoparticles. Compounds that can be used for the hydrophilic segment of nanoparticles that are used for drug delivery include polyethylene glycol (PEG), polyvinylpyrrolidone (PVP), polyacrylamide (PAM), polyvinyl alcohol (PVA), polyethylene oxide (PEO), and polyethyleneimine (PEI). Hydrophobic components can be derived from polylactide (PLLA), polylactic glycolate (PLGA), polybenzylaspartic acid (PBLA), polyglycolide (PGA), polycaprolactone (PCL), polyaspartic acid (PAsp), and polyglutamic acid (PGlu) [166]. Certain nanomaterials are more suitable for the delivery of genetic material, such as poly-L-arginine (ARG), which can be used as part of a layer-by-layer nanoparticle formulation to deliver gene-based therapies. A prior study combining hydroxyapatite with ARG in alternating layers of nanomaterials and siRNA for *SPARC* knock-down achieved gene silencing for up to two weeks in murine primary conjunctival fibroblast cells [188]. Similarly, these nanomaterials can be used to synthesize and add chemical modifications to several types of nanoparticles for ocular therapy delivery, including nanomicelles, lipid-based liposomes, polymer-based nanospheres and dendrimers, and peptide-based nanoparticles [187].

#### 3.4.2. Nanomicelles

Nanomicelles are amphiphilic molecules that spontaneously form spheres due to non-covalent interactions, with the hydrophilic heads facing outward and the hydrophobic tails facing inward, and which can deliver therapeutics to the eye, including drugs and gene vectors [11,189,190]. The hydrophobic tails of micelles can be chemically modified to increase their interaction with specific drugs [11]. Nanomicelles can be subdivided into natural nanomicelles, which include chitosans, albumin, and hyaluronic acid, and synthetic nanomicelles [11]. Natural nanomicelles are often preferred due to their greater biocompatibility and reduced toxicity, as well as their effective adhesion to and uptake by corneal cells. PEG nanomicelle formulations show potential toxic effects [191] when compared with natural formulations such as chitosan. However, some synthetic nanomicelles, such as PLLA, PGA, and PCL, are also biocompatible and break down into non-toxic byproducts, resulting in their FDA approval [189].

Preclinical testing in animal models, such as mice, rats, and rabbits, demonstrate the ability of nanomicelles to release therapeutics over sustained durations in both anterior and posterior segment tissues. Topical delivery of a block copolymer nanomicelle that was loaded with dexamethasone, an anti-inflammatory eye drop formulation that can be used for dry eye and uveitis, showed sustained release and increased bioavailability in the rabbit cornea compared with dexamethasone alone [192]. The commonly used cyclosporin A treatment for dry eye, delivered using different nanomicelle formulations into rabbits, showed increased bioavailability when compared with the drug alone [193,194]. Topical administration of glaucoma drugs, such as dorzolamide, indomethacin, and nimodipine, delivered using nanomicelles, also showed increased biocompatibility when compared to each drug alone [195,196,197]. Nanomicelles can be used to deliver topical therapeutics to the posterior segment tissues. Aflibercept, an anti-VEGF therapy for neovascular AMD, was encapsulated in an anti-angiogenic topical nanomicelle formulation containing PEG, PPG, and PCL components, which demonstrated greater efficacy when compared with aflibercept alone [198]. Nanomicelles are an effective method of delivery of hydrophobic therapeutics to the eye, particularly the anterior segment tissues. Liposomes may be more appropriate than nanomicelles for therapies that are hydrophilic.

#### 3.4.3. Liposomes

Lipid-based nanoparticles are between 80 nm and 10 μm in size and are made of a single or double phospholipid bilayer, with the lipid end facing the external environment, which are suitable for the delivery of both hydrophobic and hydrophilic drugs [11]. There are three categories of liposomes, including small unilamellar vesicles and large unilamellar vesicles, with a single phospholipid bilayer, and multilamellar vesicles, with more than one phospholipid bilayer [11]. One of the major advantages of liposomes is their biocompatibility due to their similarity in terms of chemical structure to the cellular phospholipid bilayer membrane, as well as their ability to deliver both hydrophobic and hydrophilic drugs [11]. The charge on the liposomal surface affects the ability of liposomes to diffuse through the ocular tissue layers. Positively charged liposomes are better able to bind to the negatively charged mucin on the corneal surface, allowing for greater penetrance of the ocular layers [199]. Subconjunctival delivery of large unilamellar liposomes that are loaded with latanoprost in the rabbit eye showed reduced intraocular pressure for a period of up to 50 days due to the large size of the vesicles, with similar efficacy to daily latanoprost eye drops [200]. Delivery of drugs to the posterior segment tissues comes with challenges of increasing the half-life of the drug as the liposome passes through the vitreous humor. Intravitreal delivery of tacrolimus that were encapsulated in liposomes showed efficacy in treating uveoretinitis in a rat model for a longer period of time of up to 14 days compared with the drug alone [201].

Liposomes with a positive charge facing the interior can also bind to a negatively charged phosphate in the DNA to create a compact structure of lipoplexes in the case of gene therapy delivery [48]. The DNA is protected from degradation in this complex and can enter a cell through endocytosis. A targeted non-viral delivery system, tested in a mouse model using a multifunctional lipid, (1-aminoethyl)imino-bi[N-(oleicylcysteinyl-1-amino-ethyl)propionamide] (ECO), to treat Leber’s congenital amaurosis type 2 (LCA2), has shown promising results [49]. Liposomes show versatility in their ability to transport both drugs and genetic material for gene therapeutic strategies. Similar to liposomes, dendrimers are another type of nanoparticle that can deliver both hydrophobic and hydrophilic drugs.

#### 3.4.4. Dendrimers

Dendrimers are branched-chain polymers with a core molecule and a range of different terminal functional moieties onto which drugs, both hydrophobic and hydrophilic, can be loaded [11]. In particular, poly (amidoamine) (PAMAM) dendrimers are a commonly tested type of dendrimer for ocular drug delivery. Studies in rabbits have shown that FITC that was delivered in PAMAM dendrimers had a greater ocular residence time in comparison with FITC in combination with another molecule that was matched by size and molecular weight with the PAMAM [202]. Delivery of PAMAM dendrimers that are loaded with pilocarpine, a muscarinic agonist, and tropicamide, a mydriatic agent, also showed sustained pupil dilation in rabbits compared with the drugs given alone [202]. In addition, PAMAM loaded with glucosamine and glucosamine 6-sulfate led to reduced scar tissue formation after glaucoma filtration surgery in a rabbit model [203]. Polymer-based vectors can also be used to condense DNA. Here, a positively charged polymer is mixed with DNA to create nano-sized polyplexes [204]. One major disadvantage of dendrimers is their potential toxicity, which has prevented FDA approval for use in ocular therapy so far [205], and further research to increase their safety is needed. Another type of nanoparticles that may be used for ocular therapy delivery are peptides, which are biocompatible and not synthetic like dendrimers.

#### 3.4.5. Peptides

Peptide-based polymers are a biocompatible nanoparticle for both drug delivery and condensing DNA for gene delivery. This modality offers unique advantages, because it selectively targets cell receptors, disrupts endosomal membranes, and in gene therapy, it can efficiently move genetic material to the nucleus, while it also offers the ability to engineer the proteins for optimal delivery. There are two major forms of peptide polymers, which are elastin-like polypeptides and silk fibroin polymers [206,207,208]. Elastin-like polypeptides further offer the advantage of heat responsiveness, a property that can be leveraged to trigger release in response to the body temperature, while fibroin polymers form beta-pleated sheets that may allow for sustained therapeutic release [206]. Additionally, peptides trigger minimal immune responses and can be administered at higher doses [209]. Elastin-like polypeptides have mainly been used for corneal disease, and these peptides show high adsorption to contact lenses and subsequent retention, which can allow for extended release [210]. Similarly, silk fibroin polymeric hydrogels have been tested in rabbits for delivery of bevacizumab, which showed sustained delivery for up to 90 days along with biodegradation after this time [211]. Polypeptide-based delivery systems may also be used to carry gene therapies. A positively charged peptide, abundant in lysine/arginine, can form a dense, compact complex with DNA. Further progress in preclinical studies will contribute to future clinical trials to test if a similar level of efficacy and safety can be maintained in human participants. 

#### 3.4.6. Future Directions

Nanoparticles offer several advantages over other methods of therapeutic delivery, including sustained release, which reduces the need for repeated administration while also being less invasive. The drug release time is often sustained, from weeks to months, for multiple types of nanoparticles, such as micelles and liposomes, when compared with conventional eye drops [11]. Some challenges that should be addressed are drug-loading capacity, the duration of sustained release, toxicity, and ocular residence time. Future studies aim toward altering nanoparticle chemistry to extend the drug-loading capacity for even longer durations; however, this must be balanced with avoiding toxicity. Current studies examine reducing the toxicity of certain nanoparticle formulations, which can also be achieved through chemical modifications [212]. Increasing the ocular residence time of nanoparticles is also key to increasing the duration of release. Future studies can further develop novel nanoparticle synthesis methods for existing nanoparticles that are more feasible to manufacture.

Another challenge of using nanoparticles as therapeutic delivery systems is the delivery to the posterior segment tissues, such as the retina and RPE, due to the presence of multiple barriers, including the vitreous humor. Future studies adding chemical modifications to existing nanoparticles would facilitate more efficient movement of the nanoparticles through ocular barriers such as the lens and vitreous body and a more effective uptake of the nanoparticles to the posterior segment. Nanoparticles that can release drugs, gene therapies, and antibody therapies to the posterior segment would preclude immunogenic responses that are typically induced by viral vector delivery methods and invasive modes of delivery, such as via injections. A focus on non-invasive delivery methods, such as contact lenses, as an alternative to injections and implants for delivering nanoparticles, should also be a priority.

### 3.5. Contact Lenses

Contact lenses, which are normally used for vision correction and sit atop the aqueous layer of the cornea, are a potential effective method of non-invasive, topical drug delivery into the eye, particularly the delivery of nanoparticles that are loaded with therapies [11,213]. Initial studies in which contact lenses were loaded with a drug by immersion showed that they can increase the uptake of dexamethasone through the corneal layers compared with topically delivered eye drops, which show greater leakiness through the lacrimal system [214]. However, a low drug uptake and lack of sustained release were downsides to immersion being used as a method for drug loading [215]. To overcome this challenge, two novel approaches were developed, which included loading contact lenses with nanoparticles carrying the drug and molecular imprinting [215,216]. Molecular imprinting involves the formation of non-covalent interaction sites on the monomeric form of a molecule to be polymerized for the creation of a contact lens. These sites make drug loading more conducive in the contact lens and reduce drug loss [217]. Contact lenses composed of poly-2-hydroxyethyl methacrylate (p-HEMA) hydrogels and loaded with lidocaine showed release over a period of up to 8 days.

Contact lenses as a delivery system for ocular diseases are a new potential treatment method that has not yet been extensively studied. They would provide a non-invasive alternative to ocular injections and implants and a potentially more sustained delivery method than eye drops. Researchers tested contacts with steroid dexamethasone in rabbits with uveitis and macular edema, which showed that the lenses provided 200 times more medication to the eye than eye drops and were as effective as injections at preventing retinal damage [218]. There is also potential for using this treatment against glaucoma. This therapeutic approach for retinal disease is still under proof-of-concept investigation and not yet ready for clinical study. To date, only one type of contact lens drug delivery system has received FDA approval. ACUVUE^®^ Theravision™ contact lenses with Ketotifen by Johnson & Johnson Vision Care, Inc., provide direct delivery of the antihistamine Ketotifen for quick and long-lasting treatment of itchy eyes due to allergies. The FDA approval of the first drug-eluting contact lenses for allergy treatment demonstrates the exciting potential of this delivery system for treating other ocular conditions and the need for further research.

#### Future Directions

The duration of release and the compatibility between the contact lens and drugs are the main challenges of this approach that need improvement. A potential method for maintaining the delivery of drug-eluting contact lenses may utilize eye drops for refilling the drug content in the contact lenses. Some of the challenges associated with nanoparticles are similar to the issues with developing a contact lens-based therapeutic release system. Optimizing the polymer chemistry could contribute to increasing the duration of release and would ensure more effective drug loading. There is still a great deal of research to be carried out on this delivery method, as it has not yet been investigated in-depth for ocular conditions other than those related to the cornea. Improving barriers for the delivery of therapies to the posterior segment could provide opportunities to use contact lenses as a method of delivering gene therapies in lieu of more invasive methods, such as the intravitreal and subretinal injections.

### 3.6. Ocular Implants

Intraocular implants are a delivery system designed for sustained drug release that reduce the need for multiple treatments. Non-biodegradable implants are typically surgically inserted intravitreally via an incision in the pars plana region posterior to the cornea and anterior to the retina, and they can be made from a range of materials, such as PVA, ethylene vinyl acetate (EVA), and polysulfone capillary fiber (PCF). Implants are epiretinally inserted so as to face the inner retinal layers, including the RGC layer. This mode of delivery is often used for implant devices, which are larger than nanoparticles carrying drugs or AAV vectors carrying gene therapy products [219]. The epiretinal insertion of implants also allows the device to be in contact with inner retinal neurons that are often the only layers that are left in the late stages of many diseases, such as RP [220].

A port delivery system (PDS) is a type of permanent, non-biodegradable implant that can be inserted into the eye for slow release over a period of time, with a mechanism to refill the therapeutic [221]. The system consists of four different parts: the extrascleral flange that sits outside the sclera, a septum made of a self-sealing silicone for taking up therapeutic refills, a storage region for the therapy, and a titanium release control element facing the vitreous body [221]. The drug, ranibizumab, a recombinant human monoclonal antibody raised against VEGF-A and used for the treatment of neovascular AMD, is chemically stable in the vitreous body and is stable at ocular temperatures. Therefore, ranibizumab was tested for delivery using a PDS. The system received FDA approval in 2021; however, it was recalled in 2023, due to a high frequency in cases of septum dislodgement [222]. Another type of non-biodegradable polymeric implant device containing cells on a scaffold that release ciliary neurotrophic factor (CNTF) and is currently being evaluated for the treatment of Macular Telangiectasia Type 2 recently completed phase III clinical trials, and results are not yet posted [223]. One issue that arises with non-biodegradable implants is the need to perform another procedure to remove the implant, which can be solved with biodegradable implants that dissolve naturally.

Biodegradable implants, typically made from materials such as polylactic acid (PLA), polycaprolactone (PCL), poly(lactic-*co*-glycolic-acid) (PLGA), and polyglycolic acid (PGA), are more biocompatible and dissolve within ocular tissue over time compared to non-biodegradable implants [11,220]. Several biodegradable implants have received FDA approval for different ocular conditions, such as Ozurdex for macular edema, Dexycu for post-operative inflammation, and Durysta for intraocular pressure in open-angle glaucoma/ocular hypertension. The cost of production and regulatory requirements are limitations of biodegradable implants, given the complexity of designing biocompatible devices. The main challenge of biodegradable implants that needs addressing is a potential uncontrolled release of the remaining drug load when degradation of the implant reaches a critical point [220]. Further investigation of the challenges faced by ocular implants is needed to improve patient treatment and outcomes.

#### Future Directions

Mechanical inefficiencies, invasiveness, a lack of biodegradability in permanent implants, along with the potential for excessive drug release upon the breakdown of biodegradable implants together pose challenges that must be overcome for the success of implants in clinical trials. The main challenge of non-biodegradable implants is invasiveness, since several surgical procedures are required to place the implant, remove the implant upon drug expenditure, and possibly insert a new implant if any of the components no longer function. In particular, preventing mechanical failure of parts that are responsible for the control of drug release within the implant is a major challenge, owing to the small size of the implant delivery system, and this must be addressed in future studies. Development of a method to refill implants without removal and prolonging drug release are necessary for the long-term success of this delivery method. More research into the breakdown of biodegradable implants is necessary for improving the controlled and prolonged release of therapies, while additional studies to improve existing methods of synthesis for biodegradable implants are needed to reduce manufacturing costs and increase availability in the clinic.

## 4. Summary

The need for effective therapeutics to treat ocular diseases has driven the development of numerous approaches for treating disease pathogenesis. This review focused on providing a comprehensive overview of current therapies and therapeutic delivery methods that are under investigation for ocular diseases, comparing the advantages and disadvantages of each method. While each therapeutic approach has unique benefits and challenges, all of them show potential as a treatment for ocular disease, as demonstrated by the numerous ongoing clinical trials. From targeted mutation-specific strategies such as gene editing/silencing therapy to ocular prosthesis devices that can simulate vision, these therapeutic strategies all show potential to be treatments in the future or already have some form of FDA approval.

The major gene therapies being researched are gene augmentation therapies focusing on delivering a normal gene of interest in patients with mutations in that gene, mutation-agnostic modifier therapies, gene editing techniques using the CRISPR system, and gene silencing [9,12]. Gene therapies show great efficacy in treating retinal diseases in patients, as demonstrated by the success of Luxturna and the clinical trials that are currently underway for treating various forms of retinal degeneration, such as RP and AMD. However, gene therapies have more limited efficacy in polygenic diseases and at advanced disease stages when the primary cell type that is affected in a given disease has fully degenerated. The route and method of delivering therapeutics plays a paramount role in the effectiveness, immune response, and side effects of therapies. Most therapies targeting diseases that affect the posterior segments are delivered via invasive injections into the ocular tissue. Additionally, viral vectors are commonly used therapeutic modalities for treatment of ocular diseases. The most commonly used viral vector for ocular gene therapy is the AAV vector, due to its balance of efficacy, safety, and immunogenic response when compared with the Adenovirus, which generates strong immunogenic responses, and the lentiviral vectors, which are derived from viruses that integrate into the host genome [123,124,145,147].

Antibody therapies do not require a viral vector system for delivery and are a commonly used treatment for neovascular retinal diseases, particularly anti-VEGF treatments, such as aflibercept, bevacizumab, and faricimab [10,43,224]. However, the disadvantage of antibody therapies is that they have a limited sustained effect that requires multiple repeat injections to maintain efficacy and target the symptoms of disease and not the pathological causes. Future studies must take into consideration the method for delivering these therapies. Implants, such as the PDS for bevacizumab and the CNTF-releasing cell therapy encapsulated in an implant that is in clinical trials [223], can improve the sustained delivery of therapies into ocular tissue; however, they are invasive and can sometimes require several surgeries.

Cell therapies are currently being researched to treat both heritable diseases in the advanced stages, as well as diseases resulting from acute injury, such as corneal burns, in cases with limited viable cells. The main cell types that are used for cell therapies are LSCs and MSCs, both of which are primarily used for treatment of corneal injuries, as well as hESCs and iPSCs that are used for the generation of RPE, RGC, and photoreceptor precursor cell therapies [57,85]. Preclinical studies are showing favorable results, with a clinical trial underway for the use of retinal stem and progenitor cells for the treatment of AMD (NCT05187104); however, there are currently no FDA-approved treatments for clinical use. Cell therapies are limited by differentiation and integration into the host tissue for normal functioning cells. 

In addition to molecular and cell therapies for ocular disease, prostheses are also another area that is gaining traction and showing great potential, with FDA approval to treat ocular diseases. Prostheses include the IOL and the keratoprostheses for treating anterior segment diseases, such as cataracts and corneal injuries, as well as the retinal prosthesis to treat posterior segment diseases, such as RP and AMD. However, prostheses come with a risk of post-operative complications such as infections or dislodgement, as well as a long rehabilitation. Future research must formulate strategies for overcoming these limitations in order to improve treatment outcomes in patients.

A common delivery modality for ocular therapeutics to treat anterior segment diseases, such as dry eye and glaucoma, is through eye drops [173,174,177]. The major disadvantage with eye drops is the leakage through the lacrimal system, an issue that can be resolved through the use of nanoparticle formulations. The nanoparticles that are currently being tested for ocular therapeutic delivery include nanomicelles, liposomes, dendrimers, and peptides, which greatly increase the ocular residence time for therapeutics while also allowing for sustained release of therapeutics [11,225]. Contact lenses loaded with therapeutics that are encapsulated in nanoparticles have a particularly long duration of release but are limited in their drug compatibility and have not yet been investigated for most ocular conditions.

Combining different types of therapies may address the limitations of a single therapeutic for treating disease. Nanoparticle formulations are already being evaluated with eye drops, contact lenses, and prostheses to produce a more sustained effect of therapies for anterior segment diseases and reduce infections associated with prostheses. Combination therapeutics that combine molecular and cell therapies or molecular therapies with device delivery could be potentially powerful and more robust future treatment strategies for retinal diseases. Modifier therapies that are combined with gene augmentation therapies would address perturbations in the causative mutations, as well as reset important cell homeostasis pathways. Another potential combination therapy is the use of both cell and gene therapy to stabilize the transplanted cells and increase treatment efficacy. 

Research is currently underway to use Google Glass as a potential vision aid for both magnifying information in the visual field, as well as improving contrast sensitivity [226,227]. Another exciting possibility for future ocular prosthesis is the development of advanced retinal implants not requiring an intact retina for patients with advanced forms of vision loss. The success of such a device is contingent on numerous factors, such as the ability of such a device to faithfully convert light information into an electrical impulse and transmit that to the thalamus and subsequently to the visual cortex [228]. The ORION cortical device that is currently being evaluated is a device that bypasses the intermediate stages of the visual pathway and transmits light information directly to the cortex [229]. Artificial intelligence (AI)-driven devices could be an innovative approach to convert light information that is detected by the implant device into a cohesive image that can then be transmitted directly to the brain to ensure that the patient can perceive an image of the visual field instead of simple light information. While the power of AI technologies is immense, equally substantial is the responsibility to avoid AI hallucinations that would provide false vision. These new areas of technological advances will give rise to and inform novel therapies for ocular diseases.

## Figures and Tables

**Table 1:** Current clinical trials and FDA-approved ocular therapies. Delivery systems and routes currently in use in clinical trials and FDA-approved treatments. Trials listed are currently active clinical trials of inherited ocular diseases, as shown on clinicaltrials.gov (accessed on 1 December 2023).

Disease	Delivery System	Delivery Location	Clinical Trial Number	FDA-Approved Therapy
Achromatopsia	AAV	Subretinal	NCT03278873, NCT02935517, NCT02599922	None
Dry AMD	Retinal prosthesis, Stem cells	Subretinal Intravitreal	NCT03392324, NCT01736059, NCT04339764, NCT04627428, NCT05187104	None
Wet AMD	Lentivirus, Monoclonal antibody, AAV, Axitinib suspension, Axitinib implant, Durasert, Soluble protein decoy, Brachytherapy	Subretinal Intravitreal Suprachoroidal Episcleral	NCT01678872, NCT04777201, NCT04832724, NCT05891548, NCT04989699, NCT05381948, NCT04423718, NCT05112861, NCT02988895	Aflibercept (Eylea), VEGF inhibitor, for intravitreal injection. Faricimab (Vabysmo), VEGF and Ang-2 inhibitor, for intravitreal injection. Ranibizumab (Lucentis, Byooviz), VEGF inhibitor, for intravitreal injection. Brolucizumab (Beovu), VEGF inhibitor, for intravitreal injection.
Geographic Atrophy Secondary to AMD	Pegcetacoplan (APL-2) C3 inhibitor, Antisense Inhibitor of Complement Factor B, AAV	Intravitreal Subcutaneous Subretinal	NCT04770545, NCT03815825, NCT04656561, NCT06018558	Pegcetacoplan (Syfovre), C3 inhibitor, for intravitreal injection. Avacincaptad pegol (Izervay), C5 inhibitor, for intravitreal injection.
Choroideremia	AAV	Intravitreal	NCT04483440	None
Diabetic Macular Edema	Triamcinolone acetonide, Ranibizumab, Anti-IL6 monoclonal antibody, Dexamethasone, Bevacizumab, Drug implant, aflibercept	Suprachoroidal Intravitreal Eye drops	NCT05512962, NCT05151744, NCT05066997, NCT05112861, NCT04469595, NCT04411693, NCT04108156, NCT04429503	Ozurdex biodegradable implant for sustained dexamethasone release. Faricimab (Vabysmo), VEGF and Ang-2 inhibitor, for intravitreal injection. Ranibizumab (Lucentis, Byooviz), VEGF inhibitor, for intravitreal injection. Aflibercept (Eylea), VEGF inhibitor, for intravitreal injection. Brolucizumab (Beovu), VEGF inhibitor, for intravitreal injection.
Diabetic Retinopathy	Stem cells, Selective integrin inhibitor, Ranibizumab implant, Aflibercept, Brolucizumab	Intravitreal Eye drops	NCT01736059, NCT05409235, NCT04503551, NCT04708145, NCT04278417	Aflibercept (Eylea), VEGF inhibitor, for intravitreal injection. Ranibizumab (Lucentis, Byooviz), VEGF inhibitor, for intravitreal injection.
Dry Eye Disease	Lipid conjugated chemerin peptide agonist, Small molecule, siRNA, rhNGF, TRPM8 agonist, synthetic peptide, Thermomechanical system	Eye drops Peri-orbital	NCT05759208, NCT05403827, NCT05310422, NCT05133180, NCT05493111, NCT05136170, NCT05467293, NCT05162261, NCT04795752	Miebo, Restasis, a cyclosporin drug, and Eysuvis, an ophthalmic suspension with loteprednol etabonate as eye drops.
Glaucoma	Human retinal pigment epithelium cell therapy, carbonic anhydrase inhibitor, prostaglandin F2 alpha analog, EP2 receptor agonist	Intravitreal Eye drops Peri-orbital	NCT02862938, NCT02390284, NCT05397600, NCT04761705, NCT03850782, NCT03868124, NCT03519386	Prostaglandins: Latanoprost, Ravoprost, Tafluprost, and Bimatoprost (implant/eye drops) as eye drops. Rho kinase inhibitors: Netarsudil as eye drops. Cholinergic agonists: Pilocarpine as eye drops. Alpha-adrenergic agonists: Apraclonidine and Brimonidine as eye drops. Beta blockers: Timolol as eye drops. Carbonic anhydrase inhibitors: Dorzolamide as eye drops. Omidenepag isopropyl (OMLONTI), EP2 receptor agonist, eye drops.
Leber Congenital Amaurosis	AAV, RNA antisense oligonucleotide, CRISPR/Cas9	Subretinal Intravitreal	NCT01208389, NCT03920007, NCT00481546, NCT00999609, NCT03913143, NCT03872479	AAV2-RPE65 (Luxturna) for subretinal injection gene replacement therapy.
Leber Hereditary Optic Neuropathy	AAV	Intravitreal	NCT02161380, NCT03293524	None
RP	Small molecule, Stem cells, Human retinal progenitor cells, Electrical stimulation, AAV, RNA Antisense oligonucleotide, Retinal prosthesis	Intravitreal Subretinal Trans corneal	NCT05392751, NCT04925687, NCT02086890, NCT02464436, NCT02556736, NCT05285618, NCT04945772, NCT04123626, NCT05158296, NCT01736059, NCT05203939	Argus II epiretinal Prosthesis System. AAV2-RPE65 (Luxturna) for subretinal injection gene replacement therapy.
X-linked RP	AAV	Intravitreal	NCT04517149	AAV2-RPE65 (Luxturna) for subretinal injection gene replacement therapy.
X-linked Retinoschisis	AAV	Intravitreal	NCT02317887	None
Usher Syndrome	Lentivirus, RNA Antisense oligonucleotide	Intravitreal Subretinal	NCT05158296, NCT02065011	None
Stargardt Disease	Equine infectious anemia virus, AAV, complement factor C5 inhibitor	Subretinal Intravitreal	NCT01736592, NCT03364153, NCT05956626	None

**Table 2:** Ocular therapeutic delivery systems. Delivery systems used to deliver therapeutics for ocular disease that include viral vectors such as AAVs, Ad viruses, and lentiviruses, as well as eye drops, nanoparticle formulations, and implants, along with the duration of release, efficacy, and toxicity associated with each system. Vg, viral genome copies; IUs, infectious units.

Name	Ocular Tissue Type	Duration	Efficacy	Toxicity
Adeno-associated virus				
AAV1	*Subretinal*: RPE [125].	Expression up to 6 months [125].	High transduction efficiency in ONL [126].	Inflammation above 5 × 10^10^ vg/eye [127].
AAV2	*Intravitreal*: Retinal ganglion cells, Müller cells, ciliary body, inner nuclear layer (INL). *Subretinal*: Mainly photoreceptors, also RPE.	Initial expression at 28 days post-injection [128]. Expression continues at 7–8 months post-injection [129].	Moderate transduction efficiency of photoreceptors and RGCs, low transduction of RPE cells [128].	Formation of AAV2 antibodies and possible retinal detachment above 8.0 × 10^10^ vg/eye [130,131].
AAV4	*Subretinal*: RPE, retina [125].	Expression up to 6 months [125].	High transduction efficiency of the ONL [128].	Inflammation observed above 4.8 × 10^10^ vg/eye [132].
AAV5	*Subretinal*: RPE, photoreceptors [125].	Initial expression starting at 14–21 days post-injection. Expression continues at 7–8 months post-injection [129].	High transduction efficiency in the retina compared with other AAVs [129].	Retinal thinning observed above 1.1 × 10^12^ vg/eye [133].
AAV8	*Subretinal*: photoreceptors.	Expression up to 6 months [125].	Low transduction efficiency in the ONL [128].	Inflammation and anti-AAV8 antibodies above a dose of 1 × 10^10^ vg/eye [130].
AAV9	*Subretinal*: Photoreceptors.	Expression up to 6 months [125].	Moderate transduction efficiency in the ONL [128].	Immunogenic responses observed at doses above 1 × 10^10^ vg/eye [134].
Adenovirus				
Adenovirus serotype 5 (AdV5)	*Subretinal*: All retinal layers except ONL, some RPE cells [135].	Expression begins ~2 weeks post-injection. Expression continues up to 6 months post-injection [136].	Low due to high prevalence of Ad5 serotypes in humans.	Triggers immune responses starting at 4 × 10^5^ IU/eye [137].
Lentiviral vectors				
Lenti-VSVG (lentiviral vectors pseudotyped in a vesicular stomatitis virus glycoprotein envelope)	*Intravitreal*: INL. *Subretinal*: RPE, photoreceptors.	Initial expression by 7 days post-injection. Expression continues 4–5 months post-injection [128].	High transduction efficiency.	Triggers immune response at 1.4 × 10^7^ IU/eye [128]. Derived from virus that can integrate into host genome.
Lenti–Mokola (Mokola envelope)	*Subretinal*: RPE.	Initial expression by 7 days. Expression continues at 3 months post-injection [128].	High transduction efficiency.	Triggers immune response at 6 × 10^5^ IU/eye [128]. Derived from virus that can integrate into host genome.
Topical				
Eye drops	Cornea, trabecular meshwork.	A few hours.	Low to moderate due to leakage through nasolacrimal system.	Concentration of the preservative, benzalkonium chloride (BAK), should be below 0.0005% to avoid toxicity to eye [138].
Nanoparticles				
Nanomicelles	Ocular anterior and poster segments.	Sustained release over several days [11].	High for delivery of hydrophobic drugs to the cornea and trabecular meshwork.	Concentration should be less than 2 mg/mL [139,140].
Liposomes	Cornea, retina (blood–retinal barrier).	Up to 3 months [11].	High efficacy for corneal and blood–retinal barrier uptake.	Low toxicity up to concentrations of 50 µg/mL [141]. Dependent on addition of chemical groups.
Dendrimers	Cornea, retina.	A few weeks to a month [11].	High corneal drug residence time. Can cross blood–retinal barrier depending on polymer chemistry [11].	Low toxicity at concentrations up to 100 μg/mL [142,143]. Dependent on chemical modifications.
Devices				
Contact lens	Cornea, conjunctiva, trabecular meshwork.	Immersion in drug: temporary duration. Nanoparticle-loaded: several days.	Moderate drug uptake through the corneal layers [11].	May cause corneal inflammation [11].
Intraocular Implants	Lens, trabecular meshwork, retina.	Sustained release over several months.	High due to intraocular localization.	Low toxicity for many biodegradable implants [11].
Retinal prosthesis	Retina.	Permanent electrical stimulation.	Moderate: limited by reduced specificity of cell targets and higher thresholds of inner retinal neurons compared with photoreceptors.	Generally considered biocompatible [90].

## Data Availability

No new data were presented.
